# Krukovine Suppresses KRAS-Mutated Lung Cancer Cell Growth and Proliferation by Inhibiting the RAF-ERK Pathway and Inactivating AKT Pathway

**DOI:** 10.3389/fphar.2018.00958

**Published:** 2018-08-22

**Authors:** Huanling Lai, Yuwei Wang, Fugang Duan, Ying Li, Zebo Jiang, Lianxiang Luo, Liang Liu, Elaine L. H. Leung, Xiaojun Yao

**Affiliations:** ^1^State Key Laboratory of Quality Research in Chinese Medicines, Macau University of Science and Technology, Taipa, Macau; ^2^Macau Institute for Applied Research in Medicine and Health, Macau University of Science and Technology, Taipa, Macau

**Keywords:** krukovine, KRAS, non-small cell lung cancer, natural products, inhibitor, RAF, ERK, AKT

## Abstract

Oncogenic activation of the KRAS gene via point mutations occurs in 20–30% of patients with non-small cell lung cancer (NSCLC). The RAS-RAF-ERK and RAS-PI3K-AKT pathways are the major hyper-activated downstream pathways in RAS mutation, which promotes the unlimited lifecycle of cancer cells and their metastasis in humans. However, the success of targeted therapy is restricted by many factors. Herein, we show a new pharmacological KRAS signaling inhibitor krukovine, which is a small molecular bisbenzylisoquinoline alkaloid, isolated from the bark of *Abuta grandifolia* (Mart.) Sandw. (Menispermaceae). This alkaloid targets the KRAS downstream signaling pathways in different NSCLC cell lines, such as H460 and A549, which are established by KRAS mutations. In the present study, we initially investigated the anti-cancer activities of krukovine in KRAS-mutated NSCLC cell lines, as well as KRAS wild type cancer cell line and normal lung cell. Results indicated that krukovine can inhibit the growth and dose-dependently inhibit the colony formation capacity and wound healing ability of H460 and A549. This cytotoxic effect is associated with the induction of cell apoptosis and G1 arrest in those cell lines. Krukovine treatment also suppressed the C-RAF, ERK, AKT, PI3K, p70s6k, and mTOR phosphorylation in H460 and A549. This finding suggests that krukovine represses the growth and proliferation of KRAS-mutated cells by inactivating AKT signaling pathway and downregulating the RAF-ERK signaling pathway. This study provides detailed insights into the novel cytotoxic mechanism of an anti-cancer compound from an herbal plant and promotes the anti-cancer potential of krukovine in NSCLC with KRAS mutation.

## Introduction

Lung cancer has long been a highly common cancer and the leading cause of cancer-related mortality globally, and its incidence is still increasing (Cancer Genome Atlas Research Network, 2012, 2014). About 85% of lung cancer cases are non-small cell lung cancer (NSCLC), and almost 30% of this proportion were diagnosed at an advanced stage (Abacioglu et al., 2005). In most patients with NSCLC, proto-oncogenes, such as KRAS (Kirsten rat sarcoma viral oncogene homolog), and the AKT (also named PKB, protein kinase B) and ERK (extracellular signal-regulated kinase) signaling pathways are constitutionally activated. Aberrant activation of these signaling pathways in cells leads to uncontrolled cell proliferation, apoptotic resistance, and other oncogenic cascades in many cancer types (Brognard and Dennis, 2002; Papadimitrakopoulou and Adjei, 2006; Dutta et al., 2014; Yip, 2015). Therefore, increasing research efforts have targeted these oncogenic signaling pathways to develop novel agents or therapeutics that will effectively treat NSCLC (Harada et al., 2014; Stinchcombe and Johnson, 2014).

Actually, effective inhibitors specific for many key constituents of the RAS-PI3K (phosphatidylinositol 3-kinase)-AKT and RAS-RAF-MEK-ERK pathways have been developed. Many of these inhibitors have been used or evaluated in clinical trials. A study involving 3,620 patients with NSCLC reported that KRAS is a prominent prognostic marker for the survival of patients with lung adenocarcinoma but ineffective for patients with lung small cell carcinoma (Brose et al., 2002). Patients with KRAS mutation show reduced progression-free survival, and the mutation has been adopted for biomarker analyses in NSCLC (De Grève et al., 2012; Yasuda et al., 2012). However, the development of RAS inhibitors, such as farnesyltransferase inhibitors, has been unsuccessful to date (Mazières et al., 2013). Although several AKT inhibitors have been developed and subjected to clinical trials for NSCLC treatment, their adverse side effects, such as severe hyperglycemia and other potential metabolic abnormalities, hinder their applications (Heavey et al., 2014; Yip, 2015). Side effects also limit the clinical use of the ERK inhibitor (Gioeli et al., 2011). In this regard, novel targeted drug therapy that can suppress these oncogenic pathways has attracted much research interest.

Given their low toxicity and high effectiveness, natural products have been studied and used worldwide recently as potential anti-cancer agents. Our current study identified krukovine, a novel anti-NSCLC compound from natural products. Krukovine is a small molecular bisbenzylisoquinoline alkaloid derived from *Abuta grandifolia* (Mart.) Sandw. (Menispermaceae). Menispermaceae is a well-known family of flowering plants serving as folk herbal medicine for various diseases, including gastrointestinal diseases, such as diarrhea, genitourinary tract diseases, and respiratory tract diseases (e.g., asthma) (Corrêa, 1984). Several compounds, such as bisbenzylisoquinolinic, morphinic, aporphinic, and oxoaporphinic alkaloids, have been isolated from the roots and leaves of this species (Thomas et al., 1997; de Lira et al., 2002; De Sales et al., 2015). Krukovine was first isolated from the bark of *A. grandifolia* (Mart.) Sandw. and showed potent anti-plasmodial activity decades ago (Steele et al., 1999). In the present study, krukovine exhibited a cytotoxic effect and inhibited the growth and proliferation of two KRAS-mutated lung cancer cell lines. Krukovine also inhibited the proliferation of these cancer cells by inducing G1 arrest and apoptosis. Krukovine downregulates the activity of phospho-C-RAF, phospho-AKT, phospho- p70s6k, phospho-mTOR, and phospho-ERK and modulates the PI3K-AKT-mTOR and RAF-ERK signaling pathways. Krukovine may be an alternative candidate for the development of combined targeted therapy against the abnormal expression of RAS oncogenic downstream signaling pathways in NSCLC.

## Results

### Krukovine Shows a Cytotoxic Effect Toward KRAS-Mutated Cells

**FIGURE 1:**
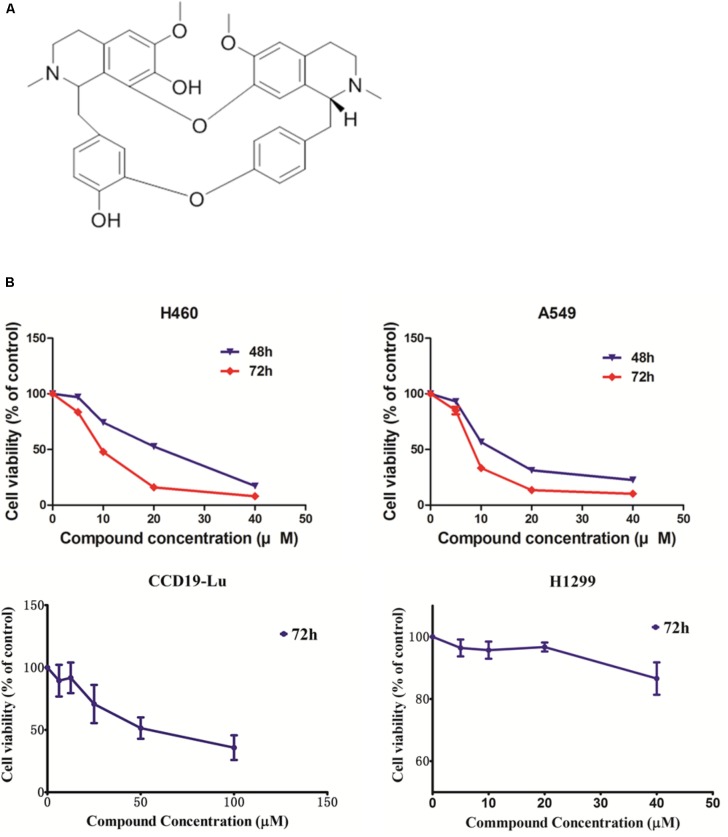
**(A)** The structure of krukovine. **(B)** Cytotoxic effect of krukovine. Cancer cell lines A549 and H460 were treated with varying concentrations of krukovine and detected by MTT assay after 48 or 72 h. Cancer cell lines H1299 and normal cell CCD19-Lu were treated with varying concentrations of krukovine and detected by MTT assay after 72 h.

To evaluate the potential anti-cancer effect of krukovine (**Figure 1A** shows the chemical structure), we subjected the KRAS-mutated cell lines H460 and A549 to cytotoxicity tests. These cell lines were treated with krukovine at 0, 5, 10, and 20 μM for 48 or 72 h. Results showed that krukovine inhibited the growth of H460 and A549 in a time-dependent manner, while have less cytotoxicity effect on non-KRAS mutation lung cancer cell line H1299 and normal lung cell CCD19-Lu (**Figure 1B**). IC_50_ values revealed the potent cytotoxicity of krukovine to KRAS-mutated cancer cells, as summarized in **Table 1**. The IC_50_ values were much lower in the H460 and A549 cell lines treated with krukovine for 72 h (9.80 ± 0.13 and 8.40 ± 0.37 μM, respectively) than in those treated for 48 h (19.89 ± 0.19 and 13.69 ± 0.15 μM, respectively).

**Table 1:** IC_50_ of KRAS-mutated cell lines after treatment with krukovine.

Cell line	IC_50_ (μM)		KRAS mutation states
	48 h	72 h	
H460	19.89 ± 0.19	9.80 ± 0.13	Q61H
A549	13.69 ± 0.15	8.40 ± 0.37	G12S
H1299	–	>40	WT
CCD19-Lu	–	56.34 ± 6.56	WT

*IC_*50*_ is presented as mean ± SEM. CCD19-Lu is normal cell*.

### Krukovine Inhibits Cell Colony Formation and Wound Healing Ability in H460 and A549 Cells

**FIGURE 2:**
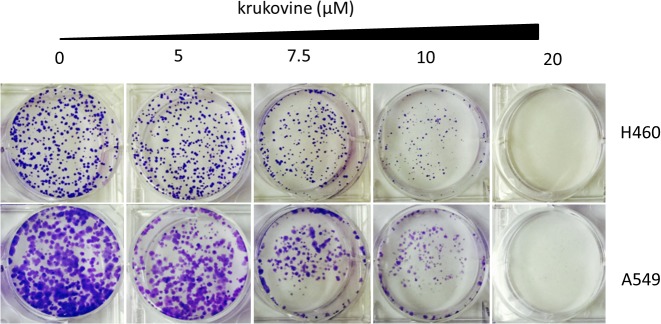
Colony formation of KRAS-mutated cells treated with krukovine (0, 5, 7.5, 10, and 20 μM) and monitored for 14 days. Representative photomicrographs of crystal violet-stained colonies were depicted.

**FIGURE 3:**
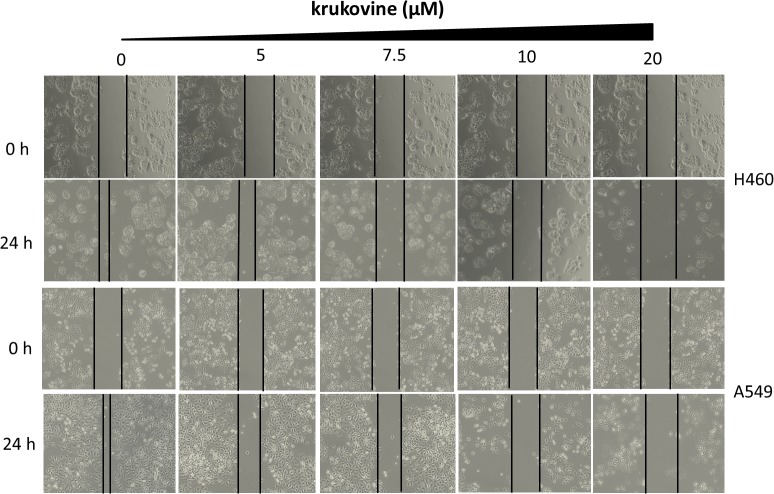
Scratch wound healing assay of KRAS-mutated cells treated with krukovine (0, 5, 7.5, 10, and 20 μM) and monitored for 24 h.

Long-term colony formation assays of H460 and A549 cells verified the growth inhibiting effect of krukovine. Krukovine significantly inhibited the colony formation capacities (**Figure 2**) and wound healing ability (**Figure 3**) of the H460 and A549 cells in a dose-dependent manner.

### Krukovine Significantly Induces Apoptosis in H460 and A549 Cells

**FIGURE 4:**
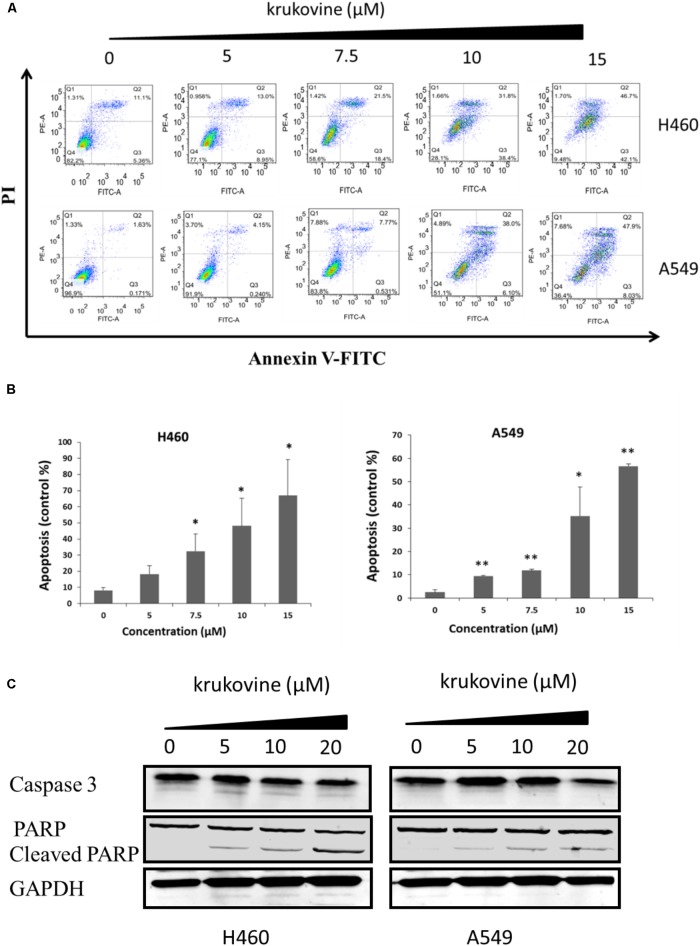
Krukovine-induced cell apoptosis in H460 and A549 cells. Two cells were treated with krukovine at different concentrations (0, 5, 7.5, 10, and 15 μM) for 48 h. **(A)** Cell apoptosis was measured by flow cytometry using Annexin V-FITC/PI staining. **(B)** Statistical analysis of the cell apoptosis rate at 48 h. All data are presented as mean ± SEM (*n* = 3, ^∗^*p* < 0.05, ^∗∗^*p* < 0.01). **(C)** Krukovine inhibits caspase-3 expression while increases PARP expression level.

To explore the anti-cancer properties of krukovine, we measured the level of cell apoptosis by flow cytometry using Annexin V-FITC/propidium iodide (PI) staining. The results are shown in **Figure 4**. Krukovine caused limited apoptosis in H460 and A549 cells in low dosage. With increased treatment dosage, the cells experienced extensive apoptosis. Krukovine inhibits caspase-3 expression while increases cleaved PARP (poly ADP ribose polymerase) expression level. This result indicated that cell apoptosis induction also contributes to the krukovine-mediated inhibition of H460 and A549 cell proliferation.

### Krukovine Induces Cell Cycle Arrest at the G1 Phase in H460 and A549 Cells

**FIGURE 5:**
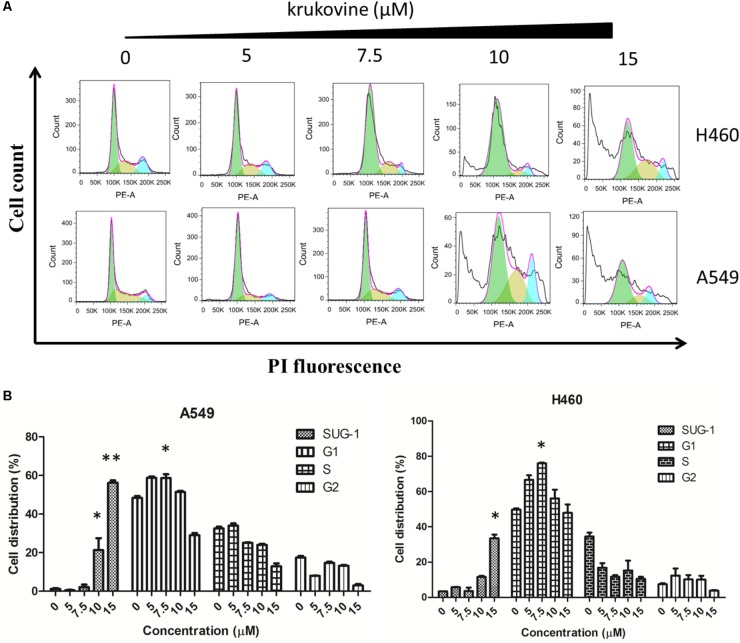
Krukovine-induced cell cycle arrest in H460 and A549 cells. Three cells were treated with krukovine at different concentrations (0, 5, 7.5, 10, and 15 μM) for 48 h. **(A)** Cells were stained with PI, and cell cycle arrest was detected by flow cytometry. **(B)** Statistical analysis of the cell apoptosis rate at 48 h. All data are presented as mean ± SEM (*n* = 3, ^∗^*p* < 0.05, ^∗∗^*p* < 0.01).

To explain the decreased cell viability, we treated H460 and A549 cells with krukovine, and their cell cycles were detected by flow cytometry through PI staining. **Figure 5** show that krukovine induced a moderate accumulation in the G1 phases and a reduction in the sub-G1 phase.

### Krukovine Inhibits the RAF-ERK Pathway and Inactivates AKT

**FIGURE 6:**
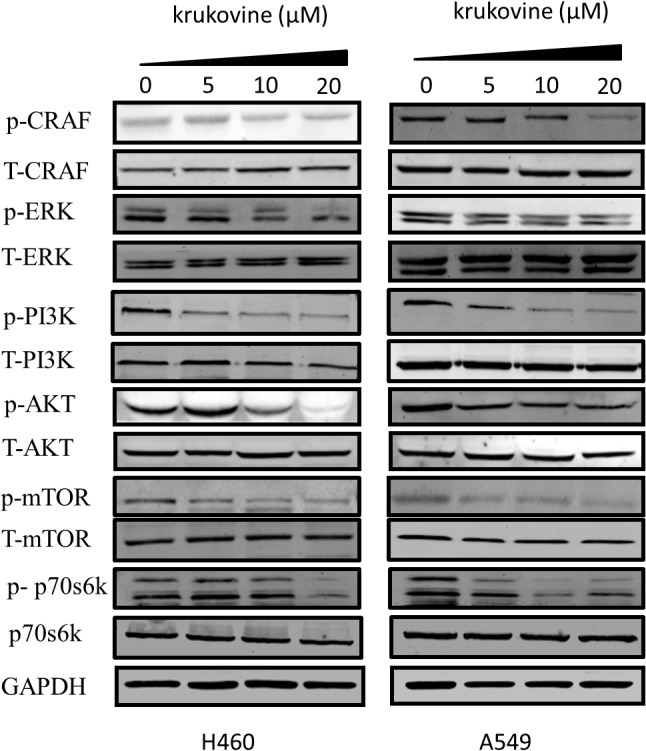
Krukovine inhibits the KRAS signaling pathway. The effect of krukovine treatment mediates the inhibition of signaling in two KRAS-mutated lung cancer cell lines. Untreated cells were used as control. A representative of at least three independent experiments for each cell line is shown.

We next evaluated the effect of krukovine on mediating the PI3K-AKT and RAF-MEK-ERK signaling pathways. These two pathways can be activated by KRAS (Turke et al., 2012; Tomasini et al., 2016). The expression levels of phospho-C-RAF, phospho-AKT (Ser473), phospho-ERK (Thr202/Thy204), phospho-PI3K, phospho-p70s6k, phospho-mTOR, total-C-RAF, total-ERK, total-p70s6k, total-mTOR, total-PI3K, and total-AKT were determined by Western blot. Results indicated that krukovine decreased the levels of phospho-C-RAF, phospho-AKT, phospho-p70s6k, phospho-mTOR, phospho-PI3K, and phospho-ERK but did not significantly affect the levels of total-C-RAF, total-ERK, total-p70s6k, total-mTOR, total-PI3K, and total-AKT (**Figure 6**). These data suggests that krukovine exerts its growth-suppressing and proliferation-inhibiting effects by regulating KRAS downstream signaling pathways.

## Discussion

The RAS-RAF-ERK and PI3K-AKT-mTOR pathways are two main downstream signaling pathways involved in the KRAS genes (Gioeli et al., 2011; Tomasini et al., 2016; Matikas et al., 2017). About 20–30% KRAS mutation (Prior et al., 2012; Stephen et al., 2014), 50–70% overexpression of phosphorylated AKT (Yip, 2015), and 70% activated ERK expression (Heavey et al., 2014) were found in patients with NSCLC. The relatively limited subset of NSCLC carrying these genetic mutations should be effectively treated by mediated target therapy, such as using RAF, ERK, and AKT inhibitors. Unfortunately, most patients with NSCLC do not harbor these genomic events, and the 5-year survival rate remains unsatisfactory (Nussinov et al., 2018). Moreover, the side effects of the target inhibitors have hindered their clinical use (Gioeli et al., 2011; Heavey et al., 2014; Yip, 2015).

For many years, natural products have been considered as potential resources for novel drug discovery. We identified a new class of small-molecule from herbs that exhibit effects on directly inactivating AKT signaling and downregulating RAF-ERK signaling pathway. In this study, we initially investigated the potential of krukovine to suppress the growth and proliferation of KRAS-mutated NSCLC cell lines. H460 and A549 cells, which contain different codons of KRAS mutation, have served as typical types of KRAS-mutated NSCLC cell lines widely used as *in vitro* model systems. Krukovine exerts cytotoxic and anti-proliferative effects on H460 and A549 cells.

We also found that the activities of pivotal proteins, such as AKT, RAF, and ERK, in the RAS-PI3K-AKT-mTOR and RAS-RAF-MEK-ERK signaling pathways are inhibited by krukovine in NSCLC cells. The interaction of the activated RAS-RAF-MEK-ERK pathway with multiple effectors can regulate cell growth, cell differentiation, and apoptosis (Cully and Downward, 2008; Montagut and Settleman, 2009). Phosphorylation of the ERK protein is a key component of the RAS-RAF-MEK-ERK downstream signaling pathway. Phosphorylated ERK translocates to the nucleus and then causes gene expression changes and mediates the activities of various transcription factors (Roberts and Der, 2007). The PI3K-AKT-mTOR signaling pathway plays an important role in cell growth, cell proliferation, angiogenesis, and cell survival; these processes determine treatment resistance against systemic chemotherapy and radiation (Pal et al., 2008). AKT is a crucial factor in this pathway. Phosphorylation of AKT downregulates various downstream substrates, such as Bad, and can result in malignant transformation (Chang et al., 2003). During cancer cell proliferation, AKT phosphorylation can accumulate the cyclin D1 protein and also prevent the release of calcium from the mitochondria and hence avert cell apoptosis (Diehl et al., 1998). Meanwhile, inactivating AKT can inhibit the PI3K-AKT-mTOR signaling pathway and achieve a tumor-suppressive effect. In this case, the feedback activation of AKT importantly participates in the unsatisfactory clinical results of several RAS downstream pathway inhibitors in cancer treatment (Sun et al., 2005; Wei et al., 2015). In the clinics, up to 45% of patients with NSCLC show increased AKT expression (Okudela et al., 2007; Spoerke et al., 2012). In our present study, krukovine induced G1 arrest and apoptosis in H460 and A549 cells; such effects can lead to cell growth inhibition. This action can be associated with the inhibitory effect of krukovine by AKT phosphorylation and RAF-ERK pathway downregulation, which can lead to cancer cell death (Cully and Downward, 2008; Pal et al., 2008; Montagut and Settleman, 2009).

The RAF-ERK and PI3K-AKT pathways are the two major hyper-activated downstream pathways in RAS mutation; these pathways promote the uncontrolled growth of abnormal cells and their metastasis in humans. These signaling pathways have been identified as promising targets in cancer therapy in recent years (Asati et al., 2016). However, the success of targeted therapy can be limited by the developed resistance of cancer cells through the mutation of target kinases, redundancy in signaling, feedback activation of pathway components, and compensatory activation of parallel circuits (Shamma et al., 1967). Use multi-targeting synthetic signaling pathway inhibitor to treat NSCLC has been proposed by some studies (Logue and Morrison, 2012; Cheng et al., 2014). In this light, targeting two or more constituents of the same pathway or two different pathways simultaneously, for example, AKT and ERK, has been suggested to improve the success of NSCLC-targeted therapy (Meng et al., 2010; Heavey et al., 2014).

In our study, we identified krukovine as a novel KRAS signaling inhibitor and evaluated its anti-cancer activity in NSCLC cell lines. Krukovine effectively inhibited KRAS downstream signaling and induced G1 arrest and apoptosis to exert a cytotoxic effect on KRAS-mutated lung cancer cells lines.

## Materials and Methods

### Reagents and Antibodies

Krukovine was purchased from Top Science (Shanghai, China) and dissolved in dimethyl sulfoxide (DMSO). The Annexin V/PI staining kit was produced by BD Biosciences (San Jose, United States). The antibodies for Western blot are as follows. Primary antibodies against phospho-C-RAF, total-C-RAF, phospho-ERK(Thr202-Thy204), total-ERK, phospho-AKT(Ser473), phospho-PI3K, total-PI3K, phospho-p70s6k, total-p70s6k, phospho-mTOR, total-mTOR, caspase-3, PARP, and GAPDH were produced by CST (Cell Signaling Technology, United States). The primary antibody against AKT was produced by Santa Cruz Biotechnology (Santa Cruz, United States). Fluorescein-conjugated goat anti-rabbit and -mouse secondary antibodies were produced by Odyssey (Belfast, United States).

### Cell Lines and Cell Culture

The KRAS mutant NSCLC cell lines used in this study (H460 and A549) were purchased from the ATCC (American Type Culture Collection, United States). All cells were cultured in RPMI-1640 medium containing 10% fetal bovine serum with 100 μg/mL streptomycin and 100 U/mL penicillin. Cells were cultured in an incubator with 5% CO_2_ at 37°C.

### Cell Growth Inhibition Assay

The standard MTT (3-(4,5-dimethylthiazol-2-yl)-2,5-diphenyltetrazolium bromide) assay was carried out to evaluate the cell growth inhibition effect of krukovine. In brief, H460, A549, H1299, and CCD19-Lu cells were each planted at 4 × 10^3^ cells or 3 × 10^3^ cells per well in a 96-well plate and cultured for 12 h to allow cell adhesion. Different concentrations (0, 5, 10, and 20 μM) of krukovine were applied as treatment for another 48 or 72 h. DMSO treatment served as a vehicle control. Every dosage was repeated three times, and at least three independent experiments were performed. At the end of the treatment, MTT solution (5 mg/mL) was added to each well (10 μL per well), and each plate was placed back to the incubator. After further culture for 4 h, the supernatant was carefully removed, and 100 μL of DMSO, as resolved solution, was added to each well while lightly shaking for 10 min to dissolve the MTT crystals. The absorbance was measured by a Tecan microplate reader at 570 nm and used as a reference at 650 nm. The percentages obtained from the absorbance of the treated cells divided by the absorbance of untreated cells were presented as the cell viabilities. The IC_50_ of krukovine was calculated by the GraphPad Prism 5.0 software.

### Cell Apoptosis and Cell Cycle Analysis

H460 and A549 cells were each planted on a six-well plate with a density of 1 × 10^5^ cells per well overnight. The cells were cultured for over 12 h to allow cell adhesion and then exposed to various concentrations of krukovine for 48 h. At the end of treatment, cells were harvested using trypsin, washed with PBS, and then collected after centrifugation. For cell apoptosis analysis, cells were treated with 5 μL of PI (1 mg/mL) and 5 μL of Annexin V fluorescein dye and stained for 15 min; this step must be performed away from light and at room temperature. The cells were then resuspended in 300–500 μL of Annexin binding buffer and filtered before analysis by a BD FACSAriaIII flow cytometer (BD Biosciences). The percentage of apoptotic cells was quantitatively determined. For cell cycle assay, the cells were fixed with 70% (v/v) ethanol for at least 30 min at 4°C. Thereafter, the cells were washed with PBS before treatment with 5 μL of PI (1 mg/mL). The percentages of cells at different cell cycle phases (sub-G, S, G1, and G2) were quantitatively measured by the same equipment in the corresponding process.

### Western Blot

Total-cell protein lysates and Western blot materials were prepared as follows. Then, 48 h after drug treatment, the cells were rinsed with lysed ice-cold PBS and lysed in RIPA buffer (150 mmol/L NaCl, 50 mmol/L Tris–HCl, pH 8.0, 1% deoxycholate, 0.1% SDS, and 1% Triton X-100) containing protease and phosphatase inhibitors (Roche, United Kingdom) for at least 30 min and then centrifuged at 14,000 ×*g* for 10 min at 4°C. The concentration of total protein for each sample was measured by DCTM protein assay kit (Bio-Rad). Then, equal amounts of total protein of each sample were resuspended in loading buffer and denatured for 5 min in 100°C. The total protein (30 μg) of each sample was separated by 10% SDS-PAGE and then transferred to PVDF membranes (Millipore, United States). The protein membranes were blocked by 5% non-fat milk in 1× TBST for 1 h at room temperature. The samples were incubated with different primary antibodies [phospho-C-RAF, phospho-AKT (Ser473), phospho-ERK (Thr202/Thy204), total-C-RAF, total-ERK, total-AKT, total-p70s6k, phospho-p70s6k, total-mTOR, phospho-mTOR, phospho-PI3K, total-PI3K, caspase-3, PARP, and GAPDH] at 4°C overnight. The above-mentioned primary antibodies were diluted in 1:1,000. Protein membranes were subsequently incubated with secondary fluorescent antibodies for 2 h and then washed in 1× TBST three times for 5 min each time. All secondary antibodies (anti-rabbit or anti-mouse) were diluted in 1:10,000. All membranes were analyzed by an LI-COR Odyssey scanner (Belfast, United States).

### Colony Formation Assay

H460 and A549 cells were planted into six-well plates (500 cells/well), respectively. After attachment overnight, the cells were treated with various concentrations (0, 5, 7.5, 10, and 20 μM) of krukovine, and the medium was changed every 3 days. When colony formation was visible, the medium was discharged. The colonies were washed with ice-cold PBS gently, fixed in 4% paraformaldehyde (PFA) for 15 min, and then stained with 0.5% crystal violet (20% methanol, 0.5% crystal violet, and 1% PFA in ddH_2_O) for 30 min. After the extra crystal violet was washed away and dried off, the colonies were photographed and analyzed.

### Scratch Wound Healing Assay

H460 and A549 cells were planted into six-well plates (500 cells/well), respectively. After attachment overnight, they should reach ∼70% confluence as a monolayer, then, the confluent monolayer was scratched with a 200 μL sterile pipette tip. After scratching, gently wash the well twice with medium to remove the detached cells. The cells were treated with various concentrations (0, 5, 7.5, 10, and 20 μM) of krukovine. After growing for additional 24 h, wash the cells twice with 1× PBS, take photos for the monolayer on a microscope in the same configurations.

### Statistical Analysis

Statistical analysis was carried out by GraphPad Prism 5.0 software. The results were presented as (mean ± SEM) of three individual experiments. ANOVA or Student’s *t*-test followed by Bonferroni’s test was used to compare all pairs of columns. *p*-Values <0.05 were set as statistically significant.

## Author Contributions

LLi, EL, and XY conceived the study, participated in the design and coordination of the whole study, and helped in critically revising the draft for important intellectual content. HL carried out the cell culture studies, molecular biology experiments, data collection, statistical analysis, and manuscript drafting. YW participated in the data collection and performed the statistical analysis. ZJ and FD participated in the molecular biology experiments. YL and LLu helped in critically revising the draft for important intellectual content. All authors have checked and approved the final manuscript and agreed to be accountable for all aspects of the work by ensuring that questions related to the accuracy or integrity of any part of the work are appropriately investigated and resolved.

## Conflict of Interest Statement

The authors declare that the research was conducted in the absence of any commercial or financial relationships that could be construed as a potential conflict of interest.
